# P-1286. Severe Forms Of Rickettsiosis In Comparison With Non-Severe Forms

**DOI:** 10.1093/ofid/ofae631.1467

**Published:** 2025-01-29

**Authors:** Fatma Hammami, Khaoula Rekik, Fatma Smaoui, Amal Chakroun, Chakib Marrakchi, Makram Koubaa, Mounir Ben Jemaa

**Affiliations:** Infectious Diseases Department, Hedi Chaker University Hospital, University of Sfax, Tunisia, Sfax, Sfax, Tunisia; Infectious Diseases Department, Hedi Chaker University Hospital, University of Sfax, Tunisia, Sfax, Sfax, Tunisia; Infectious Diseases Department, Hedi Chaker University Hospital, University of Sfax, Tunisia, Sfax, Sfax, Tunisia; Infectious Diseases Department, Hedi Chaker University Hospital, University of Sfax, Tunisia, Sfax, Sfax, Tunisia; Infectious Diseases Department, Hedi Chaker University Hospital, University of Sfax, Tunisia, Sfax, Sfax, Tunisia; Infectious Diseases Department, Hedi Chaker University Hospital, University of Sfax, Tunisia, Sfax, Sfax, Tunisia; Infectious Diseases Department, Hedi Chaker University Hospital, University of Sfax, Tunisia, Sfax, Sfax, Tunisia

## Abstract

**Background:**

Rickettsiosis, a relatively benign disease, typically manifests with fever, skin rash, and headache. However, severe forms may occur, changing therefore, the prognosis of the disease. We aimed to study the clinical, biological and therapeutic characteristics of severe forms of rickettsiosis in comparison with non-severe forms.

**Methods:**

We conducted a retrospective study including all patients hospitalized for rickettsiosis in infectious diseases department between 1996 and 2022. The diagnosis was confirmed by serological tests.

**Results:**

We encountered 84 cases of severe rickettsiosis (17.8%) and 388 cases of non-severe forms (82.2%). The median age of patients with severe forms was 47[32-64] years and with non-severe forms was 37[25-53] years (p=0.24). Meningeal syndrome (38.1% vs 8.5%; p< 0.001) and vomiting (58.3% vs 41.9%; p=0.006) were significantly more frequent among severe cases. Maculopapular skin rash (57.1% vs 86.8%; p< 0.001) and arthralgia (58.3% vs 76%; p=0.001) were significantly less frequent among severe cases. Leukocytosis was significantly more frequent among severe forms (36.9% vs 20.6%; p=0.001). Elevated C-reactive protein levels (63.1% vs 66.2%; p=0.582), thrombocytopenia (51.8% vs 59.6%; p=0.19) and hepatic cytolysis (60.7% vs 69.8%; p=0.103) were noted among severe and non-severe forms, with no significant difference. Treatment duration was significantly longer among severe forms (10[7-14] days vs 7[5-10] days; p=0.016).

**Conclusion:**

The diagnosis of severe forms of rickettsiosis should be ruled out in front of meningeal syndrome and vomiting, even in the absence of maculopapular skin rash and arthralgia, especially in the presence of leukocytosis.

**Disclosures:**

**All Authors**: No reported disclosures

